# After Action Reviews of COVID‐19 response: Case study of a large tertiary care hospital in Italy

**DOI:** 10.1002/hpm.3258

**Published:** 2021-06-06

**Authors:** Sebastiano Sorbello, Eleonora Bossi, Camilla Zandalasini, Greta Carioli, Carlo Signorelli, Fabio Ciceri, Alberto Ambrosio, Alberto Zangrillo, Anna Odone

**Affiliations:** ^1^ School of Medicine Vita‐Salute San Raffaele University Milan Italy; ^2^ Department of Public Health, Experimental and Forensic Medicine University of Pavia Pavia Italy; ^3^ Health Directorate IRCCS San Raffaele Scientific Institute Milan Italy; ^4^ Department of Clinical Sciences and Community Health Università degli Studi di Milano Milan Italy; ^5^ Department of Hematology and Bone Marrow Transplantation IRCCS San Raffaele Scientific Institute Milan Italy; ^6^ Department of Cardiovascular Anesthesia IRCCS San Raffaele Scientific Institute Milan Italy

**Keywords:** after action review, COVID‐19, hospital management, SARS‐CoV‐2, strategic reorganization

## Abstract

**Background:**

After‐Action Reviews (AARs) are management tools used to evaluate the response to public health emergencies at the national and subnational level. Aim of this study is to apply available AAR models to assess and critically appraise COVID‐19 response of San Raffaele Scientific Institute, a large university hospital in Milan, Italy.

**Methods:**

We designed an AAR based on the key‐informant interview format, following the methodology proposed by the 2019 World Health Organization Guidance for AAR. After systematic assessment of the hospital reorganization, we conducted 36 semi‐structured interviews to professionals with executive, clinical, technical and administrative roles. We designed an ad‐hoc questionnaire exploring four areas: (i) staff management; (ii) logistics and supplies; (iii) COVID‐19 diagnosis and clinical management; (iv) communication.

**Results:**

Overall, the hospital response was evaluated as effective and sufficiently prompt. Participants stressed the relevance of: (i) strong governance and coordination; (ii) readiness and availability of healthcare personnel; (iii) definition of a model of care based on a multidisciplinary approach. Challenges were reported for communication management and staff training.

**Conclusions:**

This study is one of the first applications of the AAR to the COVID‐19 response in hospital settings, which can be successfully adapted or scaled up to other settings in order to implement preparedness strategies for future public health emergencies.

## 1 BACKGROUND

After‐Action Reviews (AARs, BOX I) are structured, qualitative reviews of the actions taken to respond to public health emergencies^1^ and have been identified as one of the most useful tools to assess how preparedness systems perform,^2^ so as to achieve collective learning and continuous operational improvement in healthcare delivery.^3^ National and international health authorities, including the World Health Organization (WHO) and the European Centre for Disease Prevention and Control (ECDC) have recently suggested to apply AARs to assess public health responses to COVID‐19 in different settings.^4^ ^,^ ^5^ ^,^ ^6^

Italy was the first country in Europe to be extensively hit by the 2019 Coronavirus Disease (COVID‐19) emergency and among those experiencing the heaviest burden at the very early stages of the pandemic.^7^, ^8^ The first case of autochthonous transmission of Severe Acute Respiratory Syndrome Coronavirus 2 (SARS‐CoV‐2) was reported on 21 February 2020 in Codogno, in the Lombardy Region.^9^ Since then, the Lombardy region reported a massive outbreak escalation with 62,153 confirmed COVID‐19 cases and 11.377 deaths, accounting for, respectively, 37% of cases and 53% of deaths in the country, in the period 21 February‐15 April.^7^, ^10^

The outbreak placed an enormous strain on health systems and stretched hospitals capacity, resulting in a sharp reduction of outpatient activity and elective surgery.^11^, ^12^ Institutions and medical scientific societies shared best practices on how to reorganize activities, while minimizing the risk of infection for healthcare professionals and patients.^13^

In Milan, Lombardy, the San Raffaele Scientific Institute (SR), an Italian tertiary care university hospital with over 1200 beds and world‐wide leadership in research and clinical assistance, was frontline in the COVID‐19 public health emergency management since the very beginning. Overall, SR took care of 951 COVID‐19 patients during the first COVID‐19 epidemic wave,^14^ up to 3 May. To manage the hospital response, SR underwent a massive and rapid reorganization of clinical and surgical activities, resources and logistics, including: identification of separate pathways and dedicated departments for COVID‐19 patients, recruitment and training of healthcare and administrative personnel, drafting and introduction of operational procedures and protocols to ensure non‐deferrable clinical and surgical activities.^15^

In the midst of a public health emergency of such entity, caused by a new pathogen and a new disease, it is difficult to understand if healthcare organizations are acting efficiently, correctly and timely. In addition, the response required cross‐sector effort and collaboration that no previous event had required.^16^

Hence, it is critical to review and assess any action taken as part of the public health response, in order to capitalize on best practices and identify areas and actions for improvement. This aspect was even more relevant for SR, as the institute is located in one of the first and most affected regions in Italy.

In this context, the general aim of the current study is to conduct an AAR of the SR response to the COVID‐19 emergency. Specific aims are: (i) to adapt international guidelines to develop an AAR hospital‐based model, (ii) to apply it to SR in order to assess the preparedness during the first COVID‐19 epidemic wave, (iii) to systematically collect useful data that can fruitfully inform the planning of next waves’ response during the ongoing pandemic and improve healthcare delivery, (iv) to suggest how to share best practices and efficiently scale up the AAR methodology to other contexts and settings.

**BOX I After‐Action Review**

The AAR process and guidelines were first developed and institutionalized by the US Army in the 1970s′ as a method of providing feedbacks after collective training exercises.^17^ In such context AARs were intended as interactive discussions for unit members to decide ‘what happened, why it happened, and how to improve or sustain collective performance in future exercises’.^18^ Afterwards, the approach was adopted by the Navy and Air Force. Since then, several humanitarian organizations started adopting the AARs for organizational learning, probably because they work alongside the military in crisis response sceneries.^19^ In the past decades, the use of AARs was extended to other sectors, including the healthcare sector, as knowledge management and accountability building tool.^19^, ^20^ AAR formats can be adapted to analyse the responses and performances of health systems, institutions and facilities at national, regional and local‐level.^21^ The AAR is a component of the International Health Regulations (IHR) Monitoring and Evaluation Framework (2005).^22^ In 2019, the World Health Organization (WHO) developed a guidance for AARs^23^ and AAR toolkits^24^ in order to support health authorities in planning, preparing and conducting the reviews. According to the WHO guidance, AARs should focus on technical areas (‘pillars’) involved in the response and should be conducted within three months after the emergency event. WHO identifies four formats of AAR: debrief, working group, key informant interview and mixed‐methods. Even though AARs can be flexible in terms of purposes and methods, according to the emergency event and the assessed organizational model, all AARs should include three phases: (i) Objective observation: a structured review of responses activities; (ii) Analysis of gaps, best practices and contributing factors; (iii) Identification of areas for improvement. In 2020, the WHO and the ECDC published guidance and technical reports to support the use and the implementation of AARs focused on the public health response to COVID‐19.^5^, ^6^

## 2 METHODS

We applied and adapted the 2019 WHO guidance for After Action Reviews^23^ to conduct an AAR of SR response to the COVID‐19 first epidemic wave. The study protocol, design, implementation, data analysis, interpretation and reporting were conducted under the coordination of SR Health Directorate. The AAR was carried out in the period July‐August 2020, within three months after the end of the national lockdown on 3 May 2020.^14^ We first analysed SR COVID‐19 reorganization in terms of healthcare services, logistics, human resources management and training. Then, following the WHO AAR guidelines^23^ and the ECDC best practice recommendations,^25^ we established an AAR planning and analysis team and defined the specific objectives of the AAR: (i) to assess the functional capacity of SR to prepare and respond to the COVID‐19 outbreak; (ii) to identify best practices, bottlenecks and contributing factors; (iii) to identify fields of improvement and practical actions to be adopted in the short and long term; (iv) to provide a basis to update the SR COVID‐19 strategic preparedness and response plan.

Among the different AAR formats proposed by the WHO, and taking into account the cultural context, the complexity of the health emergency event and the resources available, we selected the ‘key informant interview’ format, identified as the longer, more in‐depth available review format.^23^ The ‘key informant interview’ format methodology includes: (i) research into background materials (peer‐reviewed literature, media reports and grey literature), (ii) semi‐structured interviews in which key‐informants are encouraged to provide honest feedback on their experiences.^26^

The list of participants was designed on the basis of SR governance structure. Professionals to be interviewed were selected both from the areas identified as pillars of the COVID‐19 response and from sectors indirectly affected by the reorganization. We identified 36 professionals with clinical, technical and administrative roles and at different career levels. The list of professional profile is reported in BOX II.

**BOX II Professional roles of participants.**

**Table:** 

	Executive Role	Operative role
**Healthcare personnel**	‐ Director of Clinical Areas and head of the Department of Cardiovascular Anesthesia	‐ Head of the Autoimmunity and Gender Medicine Unit
	‐ Coordinator of Clinical Research and head of Department of Hematology and Bone Marrow Transplantation	‐ Head of the Department of Rehabilitation and Functional Recovery
	‐ Head of clinical engineering services	‐ Five resident physicians
	‐ SR Healthcare Director	‐ Nursing Service Manager
	‐ SR Turro Healthcare Director	‐ Emergency Department Nursing Coordinator
	‐ Head of COVID‐19 Departments and of the Department of General Medicine and Advanced Care	‐ Coordinator of the Clinical Psychology Service
	‐ Director of Preventive Medicine Service	‐ Representative of the Clinical Psychology Service
	‐ Director of health professionals	‐ COVID‐19 bureau coordinator
	‐ Director of Laboratory Medicine	‐ Physiotherapist coordinator
	‐ Head of Highly Specialized Emergency Unit	‐ Physiotherapist
	‐ Director of the Postgraduate School of Hygiene and Preventive Medicine	‐ Coordinator of Budget, Flows, Outpatient Clinics and Chronic Care Area
	‐ Head of the Infectious Diseases Unit	
**Management and administrative personnel**	‐ Chief Executive Officer	
	‐ Chief Transformation Officer	
	‐ Chief Information Officer	
	‐ Chief Human Resources Officer	
	‐ Logistics and Procurement Director	
	‐ Technical Area Director	
	‐ Communication Manager	
	‐ Prevention and Protection Service Manager	
	‐ Director of Customer Service

To design the tool to carry out semi‐structured interviews, a first brainstorming was conducted within the AAR team, in order to assess the capacities in place at SR prior to the emergency event. We reviewed background materials on the following categories: organization of wards and pathways, staff management, supplies, preparedness activities, coordination mechanisms, plans and procedures. For each of these areas, we then reconstructed a timeline of key events, decisions and actions taken during the emergency. The timeline was matched with the underlying epidemiological evolution of the COVID‐19 outbreak at the regional and national level. This preliminary work was critical to identify the main areas to be reviewed in the AAR.

We then designed an *ad hoc* semi‐structured questionnaire (available as Supplementary Files‐Questionnaire S1.

For the first section of the questionnaire, respondents were asked about the perceived effectiveness of the items of four pillars:

(i) staff management (i.e., services organisation, healthcare professionals training and education, staff safety); (ii) logistics and supplies (i.e., supply of patient care equipment, personal protective equipment [PPE]); (iii) COVID‐19 diagnosis and clinical management (i.e., triage, criteria for prioritizing admissions, infection prevention and control); (iv) communication (i.e., internal communications, communications with public health authorities, use of telehealth services).

For each field of intervention, interviewees were asked to express the degree of perceived effectiveness, categorized as insufficient, sufficient, good, excellent.

Moreover, participants were asked to indicate: (i) the measures that made a significant contribution to the planning and management of the activities during the acute phase of the response; (ii) a ranking of the perceived effectiveness of the four areas of intervention (Staff, Stuff, Systems, Structure) of the ‘4S’ theory for surge capacity;^15^ (iii) the measures perceived as providing significant contribution to the planning of the recovery phase after the acute response; (iv) the perceived effectiveness of five areas of intervention (preparedness, personnel safety, readiness in the organizational response, communication during the emergency, presence of a central coordination); (v) a ranking of the perceived importance of the aforementioned five areas of intervention during public health emergencies.

Participants were also asked about their professional responsibilities during the emergency and if they had any previous experience with AARs. The questions were designed to stimulate reflections and suggestions on the most important functions under review.

After identifying the target and an initial set of candidate questions to be included in the questionnaire, we tested the acceptability of the questions through a round of piloting. Four professionals from SR Health Directorate assessed the questionnaire for readability and relevance on the basis of the background, experiences and profiles of the identified participants. Interviews were conducted in person in the period between 20 July and 3 August 2020, taking all the precautions to avoid the risk of COVID‐19 transmission among participants. A facilitator, a figure required according to the WHO guidelines,^23^ guided the interviews maintaining an impartial perspective, allowing flexibility in the discussions and facilitating the analysis of the factors that contributed to successes or failures of SR reorganization.

Descriptive statistics were used to analyse the AAR results. Analyses were also stratified by respondents’ professional profile: executive or operative roles, healthcare or management/administrative personnel, giving corresponding absolute numbers and percentages for comparison. Percentages were calculated excluding ‘Not applicable’ answers. For items that allowed multiple responses (maximum two), percentages were calculated on the total of the answers registered. For questions in which interviewees were asked to order the responses from 1 (most efficient) to 4 (least efficient), the scores were summed for each response, thus obtaining total scores that were ranked in ascending order.

In addition to the questionnaire answers, a large amount of qualitative data regarding opinions and suggestions from participants was collected during the interviews.

## 3 RESULTS

An overview of SR reorganization during the study period is available in BOX III and Table 1.

**TABLE 1:** Variations of non‐COVID services and volumes of COVID‐19 Units in the period 22 February–3 May

Reduction in Admissions to Emergency Department^a^ (%)		−62%
Reduction in scheduled surgeries (%)		−93%
Reduction in outpatient services^b^ (%)		−100%
COVID‐19 Intensive Care Units	Wards (N.)	5
	Beds (N.)	57
COVID‐19 Infectious Diseases' and Medicine Units	Wards (N.)	10
	Beds (N.)	247
COVID‐19 low Intensive Care Units	Wards (N.)	3^c^
	Beds (N.)	56
**Total COVID‐19 wards (N.)**		**18**
**Total COVID‐19 beds (N.)**		**360**

^a^ starting from an average of 204 admissions/day in previous months.

^b^ excluded non‐deferrable services (such as chemotherapy, radiotherapy, dialysis, etc.).

^c^ two wards were opened for low intensive care patients and one for patients with rehabilitation needs.

**BOX III San Raffaele Scientific Institute reorganization during COVID‐19 response**

On 22 February 2020 an Emergency Task Force was assembled to plan the reorganization of SR in response to the COVID‐19 outbreak. On 25 February, SR registered the first COVID‐19 case. The hospital underwent a massive reorganization of clinical and surgical activities, resources and logistics in order to manage the progressive increase in the number of patients identified at the triage with respiratory symptoms and requiring hospitalization. Adopting ministerial recommendations, SR separated pathways for patients with respiratory symptoms in order to limit possible contamination, both in the entrance and treatment areas.^27^ To guarantee adequate areas and personnel for clinical management of the increasing number of COVID‐19 patients, scheduled surgeries and outpatient services (excluding non‐deferrable services) were almost completely suspended.

The progressive reduction in non‐COVID‐19 activities permitted the reallocation of 360 beds for the assistance of COVID‐19 patients and the redeployment of 141 healthcare workers and 93 medical residents (in addition to the staff already employed in ED and about 90 anesthetists) among COVID‐19 Units, Emergency Department, Rehabilitation Units and bureau activities. The health care personnel were rapidly trained to use PPE and to manage COVID‐19 patients both in general Units and in ICUs.^28^ The professionals in the COVID‐19 bureau were responsible for reporting COVID‐19 cases to regional authorities for surveillance, supporting fragile patients, and scheduling the follow‐up for discharged patients. In addition, SR was identified as hub hospital for cardiovascular emergencies,^29^ following the regional reorganization of the care network for non‐COVID patients, based on the hub and spoke model.^30^

The AAR included 36 semi‐structured interviews to key‐informants (BOX II).

Of them, 27 (75%) were part of the healthcare personnel and 9 (25%) of the management/administrative personnel. With regard to career levels, 21 (58.4%) had executive positions, while 15 (41.6%) had operative ones. Less than one third of participants (30.6%) declared to be familiar with AAR and less than 10% (8.3%) to have already been involved in AARs in the past.

### 3.1 Analysis of the four pillars

The distribution of perceived effectiveness of the measures adopted within the four main pillars, overall and stratified by professional role and career level is reported in Supplementary Files‐Table S1.

**TABLE 2:** Identification of the measures that made a significant contribution to the planning and management of the activities in the acute phase of the response (each responder could mark a maximum of two responses)

	Tot: 65	Management/administrative personnel: 14	Healthcare personnel: 51	Executive: 37	Operative: 28
	N (%)	N (%)	N (%)	N (%)	N (%)
Presence of multidisciplinary teams in covid‐19 departments	27 (41.5)	6 (42.9)	21 (41.2)	15 (40.5)	12 (42.9)
Integration of clinical activity and scientific research	11 (16.9)	2 (14.3)	9 (17.6)	6 (16.2)	5 (17.9)
Availability of dedicated areas to manage the event	13 (20.0)	2 (14.3)	11 (21.6)	7 (18.9)	6 (21.4)
Systems and logistics suitable for the event	7 (10.8)	3 (21.4)	4 (7.8)	5 (13.5)	2 (7.1)
Training courses on COVID‐19 emergency management	5 (7.7)	0 (0.0)	5 (9.8)	2 (5.4)	3 (10.7)
None of the above	2 (3.1)	1 (7.1)	1 (2.0)	2 (5.4)	0 (0)

#### 3.1.1 Staff management

Overall, in the ‘Staff management’ pillar, ‘Readiness in the establishment of a Crisis Unit for the emergency management’ was perceived as the item with the highest effectiveness (Good: 34.3%; Excellent: 60.0%), followed by ‘staff management in COVID‐19 departments’ (Good 40.0%; Excellent: 40.0%) and ‘Exposed personnel management according to regional guidelines’ (Good: 38.2%; Excellent 44.1%). However, relatively large differences were recorded in the score distribution according to roles and career levels. 87.5% of management/administrative personnel rated ‘Excellent’ the ‘Readiness in the establishment of a Crisis Unit for emergency management’, compared to 51.9% of healthcare staff. 60.0% of executives rated ‘Excellent’ the ‘Exposed personnel management according to regional guidelines’ compared to 21.4% of interviewed with operative positions. Areas with least perceived effectiveness included ‘Collaboration strategies with local authorities to plan responses to shortages of healthcare personnel’ (Insufficient:15.4%; Sufficient: 26.9%).

#### 3.1.2 Logistics and supplies

The distribution of perceived effectiveness was similar for both the included questions: ‘Adequate estimation of the quantities of PPE, materials needed for patient care and personnel protection’ (Sufficient: 35.3%; Good: 44.1%; Excellent: 17.7%) and ‘Availability of PPE’ (Sufficient: 31.4%; Good: 42.9%; Excellent: 17.1%). As for the availability of PPE, the relative majority of participants with executive roles (47.6%) indicated the option ‘good’, while the relative majority of participants with operative roles (42.9%) indicated the option ‘sufficient’.

#### 3.1.3 COVID‐19 diagnosis and clinical management

Most of the feedbacks for the pillar came from healthcare personnel directly involved in this area and with the technical expertise to evaluate the response. The items with the highest perceived effectiveness were: ‘Process for COVID‐19 cases reporting to the regional health authorities’ (Good: 40.7%; Excellent: 51.9%); ‘Ability to schedule and receive COVID‐19 patient transfers from other facilities’ (Good:40.0%; Excellent: 56.7%); ‘Definition of a model of care based on levels of intensity of care and complexity’ (Good: 36.4%; Excellent: 57.6%); ‘Development of a multi‐specialist follow‐up plan for monitoring COVID‐19 discharged patients’ (Good: 39.3%; Excellent: 57.1%); ‘Emergency plan for the management and placement of deceased patients’ corpses’ (Good:47.6%; Excellent: 52.4%).

The area with the lowest perceived effectiveness was ‘Development of a protocol for active surveillance of patients with respiratory tract infections’ (Insufficient: 8.00%; Sufficient: 24.00%).

#### 3.1.4 Communication

Respondents underlined the efficiency of the ‘Collaboration with San Raffaele Directorates’ (Good: 45.7%; Excellent: 45.7%), even though with a significant difference according to the area and the role: administrative/management (Good: 22.2%; Excellent: 77.8%), healthcare personnel (Good: 53.9%; Excellent: 34.6%); executive (Good: 28.6%; Excellent: 61.9%) and operative (Good: 71.4%; Excellent: 21.4%). For the other items of the pillar, the majority of respondents reported ‘Good’ performances: ‘Appropriate signage for visitors, able to describe the appropriate precautions for infection prevention’ (41.7%); ‘Telephone numbers and other information systems (website) to provide useful information’ (40.6%); ‘Communication with health authorities to coordinate the planning of the hospital reorganization’ (44.0%); ‘Strategies for remote communication between patients and relatives’ (38.7%).

### 3.2 Identification of the measures that made a significant contribution to the planning and management of the activities in the acute phase of the response

Table 2  reports the measures perceived as having significantly contributed to the planning and implementation of SR COVID‐19 response. The ‘Presence of multidisciplinary teams in COVID‐19 departments’ ranked first (41.5%), with not substantial differences by subgroups of interest, followed by ‘Availability of dedicated areas to manage the event’ (20.0%), ‘Integration of clinical activity and scientific research’ (16.9%), ‘Systems and logistics suitable for the event’ (10.8%), ‘Training courses on COVID‐19 emergency management’ (7.7%), ‘None of the above’ (3.1%). However, we report wide differences according to professional role and career level for some items. In particular, 21.4% of management/administrative personnel chose the option ‘Systems and logistics suitable for the event’, 3‐fold higher than healthcare personnel.

**TABLE 3:** Identification of the measures that made a significant contribution to the planning of the post‐emergency reorganization (each responder could indicate only one response)

	Tot 36	Management/administrative personnel 9	Healthcare personnel 27	Executive 21	Operative 15
	N (%)	N (%)	N (%)	N (%)	N (%)
Efficient design of a plan to cope with Phase 2	20 (55.6)	6 (66.7)	14 (51.9)	12 (57.1)	8 (53.3)
Debriefing with professionals involved in the emergency response	8 (22.2)	1 (11.1)	7 (25.9)	5 (23.8)	3 (20.0)
Provide staff with a report on the progress of the emergency	1 (2.8)	0 (0.0)	1 (3.7)	0 (0.0)	1 (6.7)
Adequate recognition of services provided by staff, volunteers and outside personnel	0	0 (0.0)	0 (0.0)	0 (0.0)	0 (0.0)
Dedicated employee support program	1 (2.8)	0 (0.0)	1 (3.7)	0 (0.0)	1 (6.7)
None of the above	6 (16.7)	2 (22.2)	4 (14.8)	4 (19.1)	2 (13.3)

### 3.3 Ranking of the perceived effectiveness of the four components of the 4S theory for surge capacity

With regard to the efficiency of the items of the 4S theory for surge capacity (Staff, Stuff, Systems, Structure),^15^ staff was considered the most significant component for emergency response, followed, respectively, by structure, stuff and systems.

### 3.4 Identification of the measures that made a significant contribution to the planning of the post‐emergency reorganization

Table 3 shows the measures perceived to have the greatest positive impact in the post‐emergency (Phase 2) reorganization. The majority (55.6%) of participants indicated the ‘Efficient design of a plan to cope with Phase 2’, with slight differences by subgroup. The other options selected by respondents were ‘Debriefing with professionals involved in the emergency response’ (22.2%), ‘None of the above’ (16.7%), ‘Provide staff with a report on the progress of the emergency’ (2.8%), ‘Dedicated employee support program’ (2.8%). None of the respondents indicated the option ‘Adequate recognition of services provided by staff, volunteers and outside personnel’.

### 3.5 Impact of five key areas of intervention in public health emergencies

With regard to the ranking based on the importance in public health events of five areas of intervention (preparedness, personnel safety, readiness in the organizational response, communication during the emergency, presence of a central coordination), ‘Presence of a central coordination’ together with ‘Preparedness’ were considered the most important actions for emergency response, followed respectively by ‘staff safety’, ‘preparedness’ and ‘communication’.

Overall, almost 70% of the professionals that took part in the AAR considered the AAR an extremely useful tool; nobody assigned a score lower than 7 (score ranging from 1 ‐ not useful at all ‐ to 10 ‐ very useful) when asked to evaluate the usefulness of the tool.

## 4 DISCUSSION

We conducted an AAR of the COVID‐19 response during the first epidemic wave in a large university hospital in Milan, Italy. Our study showed that three components contributed most to the effectiveness of SR response to the COVID‐19 emergency: (i) the strong governance and coordination; (ii) the readiness and availability of healthcare personnel; (iii) the definition and implementation of an innovative model of clinical care based on levels of intensity and complexity and on a multidisciplinary approach.

Overall pooled feedback from respondents showed both strengths and areas for improvement in COVID‐19 hospital response.

Participants reported that, since the very first phases of the emergency response, the presence of a strong governance and central coordination was key. The Health Directorate promoted a constructive dialogue between the hospital directorates involved at different levels in the response, in order to plan and apply an effective emergency response. This approach allowed to efficiently implement the preparatory measures, to cope with new services requests and to ensure that the measures were promptly modified according to the needs. Prompt reconfiguration of the hospital units was reported, including the re‐allocation of medical and nursing personnel in COVID‐19 pathways. The reallocation was on a voluntary basis and SR registered a wide response from health care personnel, that expressed a high rate of willingness to be included in COVID‐19 clinical management. Multidisciplinary teams were formed promoting a profitable and constant collaboration among professionals within each team. The satisfactory results obtained by the multidisciplinary COVID‐19 model of care, pushed SR to aim at a similar approach also within the rehabilitation wards and, subsequently, in the follow‐up of discharged patients.^31^

It should be noted that, even in an emergency context, SR paid particular attention to scientific research, confirming that the synergy between clinical activity and scientific research is one of the main strengths of the institute. Of note, a biobanking project was started at a very early stage of the outbreak and will allow SR to count on one of the largest biological banks for COVID‐19 patients.

The most critical issues were reported in the areas of staff training and communication management.

With reference to the staff training, respondents stressed the lack of a structured training needs assessment. This is possibly due to the necessity to act promptly and to promote staff training methods that could have an immediate impact, for example, by pairing reallocated staff members with figures with previous specific skills in the management of patients with infectious diseases. At the same time, a slight delay in the provision of video support on dressing and undressing procedures for healthcare workers was reported.

With reference to internal communication, even though intra‐team collaboration and communication were reported as effective, there were feedbacks concerning an insufficient support and dialogue among different operating units and teams; this led to not always harmonized clinical and organizational approaches. Another critical issue in the field of communication regarded the initial difficulties in involving patients’ families, despite the efforts conducted to facilitate remote communication between hospitalized patients and their relatives.^36^ Thanks to a donation, SR has then been equipped with electronic devices which allowed the most fragile patients to communicate with their relatives.

A greater joint effort with other hospitals and health authorities was also suggested. Beyond the institutional, political and managerial difficulties, the fundamental limits of pandemic preparedness are undoubtedly represented by a limited scientific understanding and technical capacity.^32^ In this perspective, constant communication and collaboration between health authorities and healthcare providers assume even greater importance. During the response to the first COVID‐19 outbreak, SR played a crucial role of support to the Regional Healthcare System, increasing the number of beds (especially Intensive Care Unit beds) available to receive patients from other institutes and sharing medical personnel with other regional hospitals that were more distressed. Also, the lack of an integrated response plan between hospitals and community health centers placed the heavy burden of caring for COVID‐19 patients almost exclusively on the hospital healthcare components. Subsequently, respondents claimed greater attention by health authorities to the needs of hospital facilities and the introduction of new strategies to promote collaboration between hospitals and territorial primary care services.

Our study has both strengths and limitations. We only analyzed the response to the first epidemic wave of the COVID‐19 pandemic. As we acknowledge this as a limitation, we believe it was important to assess the response to the epidemic wave that placed the greatest strain on hospitals. The plan is now to conduct a second AAR to evaluate hospital response to the following epidemic phases and to compare findings. We cannot rule out participants’ answers are affected by perception bias, intended as a tendency to be subjective about the gathering and interpretation of healthcare information; however, we do expect it to be differentially distributed by professional role. Last, but not least, we do not provide here results of the debriefing sessions we conducted with all the participants and which are on‐going.

This study is one of the first application of the WHO AAR model to the COVID‐19 response in hospital settings. The main strengths of our work are: (i). strict compliance with the indications and time frames proposed by the WHO guidance,^23^ which will allow meaningful national and international comparison should the WHO AARs model be applied to other settings; (ii). the development of an *ad hoc* questionnaire tool, built on the basis of proposed models but adapted to match the characteristics of our institute; (iii). the inclusion of a complete and comprehensive list of professionals, covering the majority of executive and operative roles.

The study proved to be particularly valuable for the hospital management of SR, as it provided an assessment of the response from different perspectives. The AAR allowed the Health Directorate to propose practical steps for elimination of the bottlenecks and the introduction of new measures. Following the results of the study, the SR Health Directorate organized a discussion session and designed a follow‐up team to monitor the implementation of the proposed activities. The AAR assessment contributed to make SR able to cope in a more organized and effective way with the second wave of the pandemic, which began in the fall of 2020. It is intention of the SR Health Directorate to pursue a continuous operational improvement; the planned AAR on the COVID‐19 second wave response will help to assess the impact that the strategies, implemented following the results of this study, have had in strengthening the institute’s response. In fact, given the prolonged nature of the COVID‐19 pandemic, periodic assessments of the preparedness and response capabilities will assume critical importance. To facilitate these processes, the WHO published a guidance WHO’s Guidance for Conducting a Country COVID‐19 Intra‐Action Review’,^4^, ^5^ modelled after the WHO AAR. The guidance includes, among others, a concept note template, a facilitator’s manual, a generic presentation, a database with more than 300 COVID‐19 trigger questions and a final report template. The WHO has already supported the use of AARs concerning the responses to emerging and re‐emerging infectious disease outbreaks, environmental and natural disasters, and societal crises.^2^ Among the six WHO regions, the majority of AARs were conducted in the African region and focused on outbreaks of infectious disease (Cholera, Ebola, Dengue, Lassa Fever).^2^ Reports are available on the dedicated platform.^33^ Despite both the WHO and the ECDC having promoted the use of AARs of the public health response to COVID‐19 and provided technical guidelines,^6^ little evidence is available as for now in the literature. There is only a limited number of AAR studies mainly regarding country‐level analyses.^34^ However, it must be emphasized the importance of evaluating preparedness and operative plans even at regional and individual institution level. The application of the AAR model at local levels can guarantee a better satisfaction of local demands, stimulating local cooperation and better identifying the causes of inefficiency thanks to the proximity to the level of service provision. A systematic and multidisciplinary approach to operations assessment can also help overcome the challenges linked to an excessive fragmentation of activities and tasks within the hospital setting, the so‐called silo effect. These methodologies, in fact, not only represent a tool for organizational learning, but also increase inter‐sectoral cohesion and awareness of being part of an institution where each member can make a contribution to the decision‐making process.^35^

## 5 CONCLUSIONS

AARs are key management tools for the evaluation of the responses to public health emergencies and can strengthen health systems preparedness**.** We demonstrate the relevance of conducting AARs of COVID‐19 hospital response and we propose an *ad hoc* designed format, developed following the WHO Guidance for After Action Review AAR.^23^ As international health authorities have recommended the use of AARs of the pandemic response at different levels, we applied a sound and rigorous methodology that can be adapted and scaled up to other settings in order to inform the planning and implementation of next phases of the pandemic response and support preparedness for future public health emergencies.

## CONFLICTS OF INTEREST

The authors declare that there is no conflict of interest and this research did not receive any specific grant from funding agencies in the public, commercial, or not‐for‐profit sectors.

## ETHICAL STATEMENT

The study involved human contact, but data were anonymized and treated as aggregated.

## Supporting information

Supplementary Material S1

Supplementary Material S2

## Data Availability

The data that support the findings of this study are available from the corresponding author upon request.
