# Supplementary material for: After Action Reviews of COVID‐19 response: Case study of a large tertiary care hospital in Italy
Source: Int J Health Plann Manage. 2021 Jun 6;36(5):1758–71. doi: 10.1002/hpm.3258 (PMC8239549; doi:10.1002/hpm.3258)
Supplement: Supplementary file 1 — Supplementary Material S1 [file HPM-36-1758-s001.docx]

# Article title: After Action Reviews of COVID-19 response: case study of a large tertiary care hospital in Italy

**Journal name:** International Journal of Health Planning and Management

**Supplemental material description**:

Questionnaire for conducting an After Action Review (AAR) on COVID-19 emergency

UNIVERSITÀ VITA-SALUTE SAN RAFFAELE

FACULTY OF MEDICINE AND SURGERY

Academic Year 2019/ 2020

**QUESTIONNAIRE FOR CONDUCTING AN AFTER-ACTION REVIEW (AAR) ON COVID-19 EMERGENCY**

San Raffaele Scientific Institute

| This questionnaire is part of the study conducted for the thesis entitled "Analysis of the strategic reorganization of IRCCS Ospedale San Raffaele in the context of the COVID-19 pandemic and evaluation of the impact of the measures taken". It has been developed following the guidelines for the conduct of After-Action Reviews (AAR) proposed by the World Health Organization within the document "Guidance for After Action Review AAR" (2019). An After-Action Review is a qualitative review of actions taken in response to a public health event. It is conducted within three months of the emergency response and is intended to identify and document best practices implemented and challenges encountered during the event. It is an important management tool for assessing staff perceptions and gathering information about the main critical issues that emerged, with an aim of continuous improvement in hospital performance. Among the tools used to conduct AARs are qualitative questionnaires to be submitted to the main actors involved in the response to the event analyzed.  ***Objective***:  This questionnaire was created with the aim of identifying, through the point of view of the main protagonists, the best practices and the problems encountered by IRCCS Ospedale San Raffaele during the first epidemic wave of the COVID-19 pandemic. It also aims to identify and evaluate the mechanisms of preparedness, as well as provide insights to implement the efficiency of the response to similar events.  The questionnaire evaluates the respondents’ perception on some of the actions developed by the hospital during the emergency (personnel, resources and supplies, COVID-19 patient identification and communication), in order to assess criticalities and strengths of the organization, as well as the impact of the emergency on their professional activity.  The questionnaire is anonymous, and the data collected will be treated as aggregated. |
| --- |

- What was your professional role during the emergency?

……………………………………….……………………………………….……………

……………………………………….……………………………………….……………

……………………………………….……………………………………….……………

- Were you aware of the existence of After-Action Reviews as tools to assess response to public health events? □ Yes □ No
- Have you participated in an After-Action Review (AAR) before? □ Yes □ No

For each of the following areas of emergency response, please indicate with an “X” your perceived level of effectiveness:

0: don't know, not applicable 1: insufficient 2: sufficient 3: good 4: excellent

| **Staff management** | 0 | 1 | 2 | 3 | 4 |
| --- | --- | --- | --- | --- | --- |
| Readiness in the establishment of a Crisis Unit for emergency management |  |  |  |  |  |
| Education and training of healthcare personnel |  |  |  |  |  |
| Staff management in COVID-19 departments |  |  |  |  |  |
| Collaboration strategies with local authorities to plan responses to shortages of healthcare personnel |  |  |  |  |  |
| Exposed personnel management according to regional guidelines |  |  |  |  |  |

| **Resources and supplies** | 0 | 1 | 2 | 3 | 4 |
| --- | --- | --- | --- | --- | --- |
| Adequate estimation of the quantities of Personal Protective Equipment (PPE), materials needed for patient care and personnel protection |  |  |  |  |  |
| Availability of PPE |  |  |  |  |  |

| **COVID-19 diagnosis and clinical management** | 0 | 1 | 2 | 3 | 4 |
| --- | --- | --- | --- | --- | --- |
| Development of a protocol for the identification and management of patients with symptoms of respiratory infection |  |  |  |  |  |
| Design of separate paths for access and movement of COVID-19 patients |  |  |  |  |  |
| Development of a protocol for active surveillance of patients with respiratory tract infections |  |  |  |  |  |
| Development of a protocol for active surveillance of personnel with respiratory tract infections |  |  |  |  |  |
| Process for COVID-19 cases reporting to the regional health authorities |  |  |  |  |  |
| Ability to schedule and receive COVID-19 patient transfers from other facilities |  |  |  |  |  |
| Definition of a model of care based on levels of intensity of care and complexity |  |  |  |  |  |
| Setting up suitable protected discharge modes in support of isolation of COVID-19 patients |  |  |  |  |  |
| Development of a multi-specialist follow-up plan for monitoring COVID-19 discharged patients |  |  |  |  |  |
| Emergency plan for the management and placement of deceased patients' bodies |  |  |  |  |  |

| **Communication:** | 0 | 1 | 2 | 3 | 4 |
| --- | --- | --- | --- | --- | --- |
| Appropriate signage for visitors, able to describe the appropriate precautions for infection prevention |  |  |  |  |  |
| Telephone numbers and other information systems (website) in order to provide useful information |  |  |  |  |  |
| Collaboration with SR Directorates:   - Technical Area Directorate - Clinical Engineering Service - Pharmacy - Directorate of Purchasing and Logistics |  |  |  |  |  |
| Communication with Health Authorities to coordinate the planning of the hospital reorganization |  |  |  |  |  |
| Strategies for remote communication between patients and relatives |  |  |  |  |  |

- Which of the following made a greater contribution to the planning and management of your activities during the emergency response within SR? (Please mark a maximum of two responses)
- Presence of multidisciplinary teams in COVID-19 departments
- Integration of clinical activity and scientific research
- Availability of dedicated areas to manage the event
- Systems and logistics suitable for the event
- Training courses on COVID-19 emergency management
- None of the above

Comments:

……………………………………….……………………………………….……………

……………………………………….……………………………………….……………

……………………………………….……………………………………….……………

- Order the following emergency response areas from 1 (most efficient) to 4 (least efficient) based on perceived effectiveness during SR's strategic reorganization.

| Staff (Recruitment of staff in the facility, willingness of professionals...) |  |
| --- | --- |
| Stuff (PPE, medicines, oxygen…) |  |
| Structure (Hospital reconfiguration: new spaces and paths dedicated to COVID-19 patients, diagnostic equipment …) |  |
| Systems (Internal communication among staff members, communication with authorities outside the facility, efficiency of information systems …) |  |

- Which of the following has had the greatest positive impact in the post-emergency reorganization phase within SR? (indicate only one response)?
- Efficient design of a plan to cope with Phase 2
- Debriefing with professionals involved in the emergency response
- Provide staff with a report on the progress of the emergency
- Adequate recognition of services provided by staff, volunteers and outside personnel
- Dedicated employee support program
- None of the above
- In light of the situation addressed, give each of the following areas a score based on the effectiveness found in SR.

0: don't know, not applicable 1: very low 2: low 3: high 4: very high

|  | 0 | 1 | 2 | 3 | 4 |
| --- | --- | --- | --- | --- | --- |
| Preparedness |  |  |  |  |  |
| Personnel safety |  |  |  |  |  |
| Readiness in the organizational response |  |  |  |  |  |
| Communication during the emergency |  |  |  |  |  |
| Presence of a central coordination |  |  |  |  |  |

- Following your experience, order on a scale of 1 (most important) to 5 (least important) the following areas based on the importance of each in responding to an emergency event:

| Preparedness |  |
| --- | --- |
| Personnel safety |  |
| Readiness in the organizational response |  |
| Communication during the emergency |  |
| Presence of a central coordination |  |

- How useful do you think an After-Action Review is for professionals in your job position?

1 2 3 4 5 6 7 8 9 10
Not at all useful Somewhat useful Very useful

Comments:

……………………………………….……………………………………….……………………

……………………………………….……………………………………….……………………

……………………………………….……………………………………….……………………

……………………………………….……………………………………….……………………
