# Supplementary material for: After Action Reviews of COVID‐19 response: Case study of a large tertiary care hospital in Italy
Source: Int J Health Plann Manage. 2021 Jun 6;36(5):1758–71. doi: 10.1002/hpm.3258 (PMC8239549; doi:10.1002/hpm.3258)
Supplement: Supplementary file 2 — Supplementary Material S2 [file HPM-36-1758-s002.docx]

**Table II.** Perceived degree of effectiveness, total, by area (administrative/management and healthcare) and by role (executive and operative)

|  | **Insufficient N (row %)** | **Sufficient**  **N (row %)** | **Good**  **N (row %)** | **Excellent**  **N (row %)** |
| --- | --- | --- | --- | --- |
| **Staff management** |  |  |  |  |
| Readiness in the establishment of a Crisis Unit for emergency management* | - | 2 (5.7) | 12 (34.3) | 21 (60.0) |
| ***Area*** |  |  |  |  |
| *Management/administrative personnel** | *-* | *-* | *1 (12.5)* | *7 (87.5)* |
| *Healthcare personnel* | *-* | *2 (7.4)* | *11 (40.7)* | *14 (51.9)* |
| ***Role*** |  |  |  |  |
| *Executive** | *-* | *1 (5.0)* | *5 (25.0)* | *14 (70.0)* |
| *Operative* | *-* | *1 (6.7)* | *7 (46.7)* | *7 (46.7)* |
| Education and training of healthcare personnel* | 3 (8.8) | 6 (17.7) | 15 (44.1) | 10 (29.4) |
| ***Area*** |  |  |  |  |
| *Management/administrative personnel** | *-* | *-* | *5 (71.4)* | *2 (28.6)* |
| *Healthcare personnel* | *3 (11.1)* | *6 (22.2)* | *10 (37.0)* | *8 (29.6)* |
| ***Role*** |  |  |  |  |
| *Executive** | *1 (5.3)* | *1 (5.3)* | *9 (47.4)* | *8 (42.1)* |
| *Operative* | *2 (13.3)* | *5 (33.3)* | *6 (40.0)* | *2 (13.3)* |
| Staff management of COVID-19 departments* | 1 (2.9) | 6 (17.1) | 14 (40.0) | 14 (40.0) |
| ***Area*** |  |  |  |  |
| *Management/administrative personnel** | *-* | *1 (12.5)* | *4 (50.0)* | *3 (37.5)* |
| *Healthcare personnel* | *1 (3.7)* | *5 (18.5)* | *10 (37.0)* | *11 (40.7)* |
| ***Role*** |  |  |  |  |
| *Executive** | *-* | *3 (15.0)* | *9 (45.0)* | *8 (40.0)* |
| *Operative* | *1 (6.7)* | *3 (20.0)* | *5 (33.3)* | *6 (40.0)* |
| Collaboration strategies with local authorities to plan responses to shortages of healthcare personnel* | 4 (15.4) | 7 (26.9) | 8 (30.8) | 7 (26.9) |
| ***Area*** |  |  |  |  |
| *Management/administrative personnel** | *-* | *3 (37.5)* | *1 (12.5)* | *4 (50.0)* |
| *Healthcare personnel** | *4 (22.2)* | *4 (22.2)* | *7 (38.9)* | *3 (16.7)* |
| ***Role*** |  |  |  |  |
| *Executive** | *2 (10.5)* | *6 (31.6)* | *4 (21.1)* | *7 (36.8)* |
| *Operative** | *2 (28.6)* | *1 (14.3)* | *4 (57.1)* |  |
|  |  |  |  |  |
| Exposed personnel management according to regional guidelines* | - | 6 (17.7) | 13 (38.2) | 15 (44.1) |
| ***Area*** |  |  |  |  |
| *Management/administrative personnel* | *-* | *1 (11.1)* | *3 (33.3)* | *3 (33.3)* |
| *Healthcare personnel** | *-* | *5 (20.0)* | *10 (40.0)* | *10 (40.0)* |
| ***Role*** |  |  |  |  |
| *Executive** | *-* | *3 (15.0)* | *5 (25.0)* | *12 (60.0)* |
| *Operative** | *-* | *3 (21.4)* | *8 (57.1)* | *3 (21.4)* |
|  |  |  |  |  |
| **Resources and supplies** |  |  |  |  |
| Adequate estimation of the quantities of PPE, materials needed for patient care and personnel protection* | 1 (2.9) | 12 (35.3) | 15 (44.1) | 6 (17.7) |
| ***Area*** |  |  |  |  |
| *Management/administrative personnel* | *-* | *4 (44.4)* | *3 (33.3)* | *2 (22.2)* |
| *Healthcare personnel** | *1 ( 4.0)* | *8 (32.0)* | *12 (48.0)* | *4 (16.0)* |
| ***Role*** |  |  |  |  |
| *Executive** | *-* | *7 (35.0)* | *9 (45.0)* | *4 (20.0)* |
| *Operative** | *1 ( 7.1)* | *5 (35.7)* | *6 (42.9)* | *2 (14.3)* |
| Availability of PPE* | 3 (8.6) | 11 (31.4) | 15 (42.9) | 6 (17.1) |
| ***Area*** |  |  |  |  |
| *Management/administrative personnel* | *-* | *2 (22.2)* | *4 (44.4)* | *3 (33.3)* |
| *Healthcare personnel** | *3 (11.5)* | *9 (34.6)* | *11 (42.3)* | *3 (11.5)* |
| ***Role*** |  |  |  |  |
| *Executive* | *1 ( 4.8)* | *5 (23.8)* | *10 (47.6)* | *5 (23.8)* |
| *Operative** | *2 (14.3)* | *6 (42.9)* | *5 (35.7)* | *1 ( 7.1)* |
|  |  |  |  |  |
| **COVID-19 diagnosis and clinical management** |  |  |  |  |
| Development of a protocol for the identification and management of patients with symptoms of respiratory infection* | - | - | 11 (37.9) | 18 (62.1) |
| ***Area*** |  |  |  |  |
| *Management/administrative personnel ** | *-* | *-* | *2 (40.0)* | *3 (60.0)* |
| *Healthcare personnel** | *-* | *-* | *9 (37.5)* | *15 (62.5)* |
| ***Role*** |  |  |  |  |
| *Executive ** | *-* | *-* | *4 (25.0)* | *12 (75.0)* |
| *Operative** | *-* |  | *7 (53.9)* | *6 (46.2)* |
| Design of separate paths for access and movement of COVID-19 patients* | - | 5 (14.3) | 12 (34.3) | 18 (51.4) |
| ***Area*** |  |  |  |  |
| *Management/administrative personnel ** | *-* | *1 (12.5)* | *3 (37.5)* | *4 (50.0)* |
| *Healthcare personnel* | *-* | *4 (14.8)* | *9 (33.3)* | *14 (51.9)* |
| ***Role*** |  |  |  |  |
| *Executive** | *-* | *1 ( 5.0)* | *8 (40.0)* | *11 (55.0)* |
| *Operative* | *-* | *4 (26.7)* | *4 (26.7)* | *7 (46.7)* |
| Development of a protocol for active surveillance of patients with respiratory tract infections* | 2 (8.0) | 6 (24.0) | 9 (36.0) | 8 (32.0) |
| ***Area*** |  |  |  |  |
| *Management/administrative personnel* | *-* | *-* | *3(100.0)* | *-* |
| *Healthcare personnel** | *2 ( 9.1)* | *6 (27.3)* | *6 (27.3)* | *8 (36.4)* |
| ***Role*** |  |  |  |  |
| *Executive** |  | *2 (15.4)* | *7 (53.9)* | *4 (30.8)* |
| *Operative** | *2 (16.7)* | *4 (33.3)* | *2 (16.7)* | *4 (33.3)* |
| Development of a protocol for active surveillance of personnel with respiratory tract infections* | 2 (7.7) | 2 (7.7) | 15 (57.7) | 7 (26.9) |
| ***Area*** |  |  |  |  |
| *Management/administrative personnel** | *-* | *-* | *3 (100.0)* | *-* |
| *Healthcare personnel** | *2 ( 8.7)* | *2 ( 8.7)* | *12 (52.2)* | *7 (30.4)* |
| ***Role*** |  |  |  |  |
| *Executive** |  | *1 ( 7.7)* | *8 (61.5)* | *4 (30.8)* |
| *Operative** | *2 (15.4)* | *1 ( 7.7)* | *7 (53.9)* | *3 (23.1)* |
| Process for COVID-19 cases reporting to the regional health authorities* | - | 2 (7.4) | 11 (40.7) | 14 (51.9) |
| ***Area*** |  |  |  |  |
| *Management/administrative personnel** | *-* | *-* | *1 (33.3)* | *2 (66.7)* |
| *Healthcare personnel** | *-* | *2 ( 8.3)* | *10 (41.7)* | *12 (50.0)* |
| ***Role*** |  |  |  |  |
| *Executive** | *-* | *-* | *5 (35.7)* | *9 (64.3)* |
| *Operative** | *-* | *2 (15.4)* | *6 (46.2)* | *5 (38.5)* |
| Ability to schedule and receive COVID-19 patient transfers from other facilities* | 1 (3.33) | - | 12 (40.0) | 17 (56.7) |
| ***Area*** |  |  |  |  |
| *Management/administrative personnel** | *-* | *-* | *3 (50.0)* | *3 (50.0)* |
| *Healthcare personnel** | *1 (4.2)* | *-* | *9 (37.5)* | *14 (58.3)* |
| ***Role*** |  |  |  |  |
| *Executive** |  | *-* | *8 (44.4)* | *10 (55.6)* |
| *Operative** | *1 (8.3)* | *-* | *4 (33.3)* | *7 (58.3)* |
| Definition of a model of care based on levels of intensity of care and complexity* | - | 2 (6.1) | 12 (36.4) | 19 (57.6) |
| ***Area*** |  |  |  |  |
| *Management/administrative personnel** | *-* | *-* | *3 (42.9)* | *4 (57.1)* |
| *Healthcare personnel** | *-* | *2 ( 7.7)* | *9 (34.6)* | *15 (57.7)* |
| ***Role*** |  |  |  |  |
| *Executive** | *-* | *1 ( 5.6)* | *7 (38.9)* | *10 (55.6)* |
| *Operative* | *-* | *1 ( 6.7)* | *5 (33.3)* | *9 (60.0)* |
| Setting up suitable protected discharge modes in support of isolation of COVID-19 patients* | - | 5 (17.9) | 14 (50.0) | 9 (32.1) |
| ***Area*** |  |  |  |  |
| *Management/administrative personnel** | *-* | *-* | *1 (33.3)* | *2 (66.7)* |
| *Healthcare personnel** | *-* | *4 (16.0)* | *12 (48.0)* | *9 (36.0)* |
| ***Role*** |  |  |  |  |
| *Executive** | *-* | *3 (21.4)* | *6 (42.9)* | *5 (35.7)* |
| *Operative** | *-* | *2 (14.3)* | *8 (57.1)* | *4 (28.6)* |
| Development of a multi-specialist follow-up plan for monitoring COVID-19 discharged patients* | - | 1 (3.6) | 11 (39.3) | 16 (57.1) |
| ***Area*** |  |  |  |  |
| *Management/administrative personnel** | *-* |  | *2 (50.0)* | *2 (50.0)* |
| *Healthcare personnel** | *-* | *1 (4.2)* | *9 (37.5)* | *14 (58.3)* |
| ***Role*** |  |  |  |  |
| *Executive** | *-* | *1 (6.7)* | *5 (33.3)* | *9 (60.0)* |
| *Operative** | *-* | *-* | *6 (46.2)* | *7 (53.9)* |
| Emergency plan for the management and placement of deceased patients' bodies* | - | - | 10 (47.6) | 11 (52.4) |
| ***Area*** |  |  |  |  |
| *Management/administrative personnel** | *-* | *-* | *3 (60.0)* | *2 (40.0)* |
| *Healthcare personnel** | *-* | *-* | *7 (43.8)* | *9 (56.3)* |
| ***Role*** |  |  |  |  |
| *Executive** | *-* | *-* | *3 (25.0)* | *9 (75.0)* |
| *Operative** | *-* | *-* | *7 (77.8)* | *2 (22.2)* |
|  |  |  |  |  |
| **Communication** | - | - |  |  |
| Appropriate signage for visitors, able to describe the appropriate precautions for infection prevention | - | 11 (30.56) | 15 (41.7) | 10 (27.8) |
| ***Area*** |  |  |  |  |
| *Management/administrative personnel* | *-* | *3 (33.3)* | *2 (22.2)* | *4 (44.4)* |
| *Healthcare personnel* | *-* | *8 (29.6)* | *13 (48.2)* | *6 (22.2)* |
| ***Role*** |  |  |  |  |
| *Executive* | *-* | *7 (33.3)* | *7 (33.3)* | *7 (33.3)* |
| *Operative* | *-* | *4 (26.7)* | *8 (53.3)* | *3 (20.0)* |
| Telephone numbers and other information systems (website) in order to provide useful information* | 3 (9.4) | 8 (25.0) | 13 (40.6) | 8 (25.0) |
| ***Area*** |  |  |  |  |
| *Management/administrative personnel ** | *-* | *1 (12.5)* | *4 (50.0)* | *3 (37.5)* |
| *Healthcare personnel** | *3 (12.5)* | *7 (29.2)* | *9 (37.5)* | *5 (20.8)* |
| ***Role*** |  |  |  |  |
| *Executive** |  | *6 (31.6)* | *6 (31.6)* | *7 (36.8)* |
| *Operative** | *3 (23.1)* | *2 (15.4)* | *7 (53.9)* | *1 ( 7.7)* |
| Collaboration with SR Directorates* | - | 3 (8.6) | 16 (45.7) | 16 (45.7) |
| ***Area*** |  |  |  |  |
| *Management/administrative personnel* | *-* | *-* | *2 (22.2)* | *7 (77.8)* |
| *Healthcare personnel** | *-* | *3 (11.5)* | *14 (53.9)* | *9 (34.6)* |
| ***Role*** |  |  |  |  |
| *Executive* | *-* | *2 ( 9.5)* | *6 (28.6)* | *13 (61.9)* |
| *Operative** | *-* | *1 ( 7.1)* | *10 (71.4)* | *3 (21.4)* |
| Communication with health authorities to coordinate the planning of the hospital reorganization* | 1 (4.0) | 7 (28.0) | 11 (44.0) | 6 (24.0) |
| ***Area*** |  |  |  |  |
| *Management/administrative personnel** | *-* | *1 (20.0)* | *3 (60.0)* | *1 (20.0)* |
| *Healthcare personnel** | *1 ( 5.0)* | *6 (30.0)* | *8 (40.0)* | *5 (25.0)* |
| ***Role*** |  |  |  |  |
| *Executive** | *-* | *6 (37.5)* | *6 (37.5)* | *4 (25.0)* |
| *Operative** | *1 (11.1)* | *1 (11.1)* | *5 (55.6)* | *2 (22.2)* |
| Strategies for remote communication between patients and relatives* | 2 (6.5) | 7 (22.6) | 12 (38.7) | 10 (32.3) |
| ***Area*** |  |  |  |  |
| *Management/administrative personnel** | *-* | *3 (50.0)* | *1 (16.7)* | *2 (33.3)* |
| *Healthcare personnel** | *2 ( 8.0)* | *4 (16.0)* | *11 (44.0)* | *8 (32.0)* |
| ***Role*** |  |  |  |  |
| *Executive** | *1 ( 6.3)* | *3 (18.8)* | *5 (31.3)* | *7 (43.8)* |
| *Operative* | *1 ( 6.7)* | *4 (26.7)* | *7 (46.7)* | *3 (20.0)* |
| *The sum does not add to the total because of missing values and exclusion of not applicable category | | | | |
